# Microbiota and enteric nervous system crosstalk in diabetic gastroenteropathy: bridging mechanistic insights to microbiome-based therapies

**DOI:** 10.3389/fcimb.2025.1603442

**Published:** 2025-08-11

**Authors:** Wang Tao, Yunfeng Yu, Danni Tan, Xiangning Huang, Jiawang Huang, Chuanquan Lin, Rong Yu

**Affiliations:** ^1^ School of Traditional Chinese Medicine, Hunan University of Chinese Medicine, Changsha, Hunan, China; ^2^ School of Traditional Chinese Medicine, Guangzhou University of Chinese Medicine, Guangzhou, Guangdong, China

**Keywords:** diabetic gastroenteropathy, the enteric nervous system, gut microbiota, microbiota-ENS axis, microbial metabolites, gut hormones, neurotransmitters, microbiome-based therapies

## Abstract

Diabetes mellitus has emerged as a global public health crisis, with over half of patients experiencing gastrointestinal (GI) symptoms that exacerbate glucose fluctuations and impair quality of life. While prior research on the pathophysiology of diabetic gastroenteropathy (DGE) focused primarily on autonomic neuropathy, particularly involving the vagus nerve, recent studies have shifted toward the impairment of the enteric nervous system (ENS). As the largest autonomous neural network governing GI motility independent of central control, structural and functional abnormalities of the ENS constitute the fundamental pathological basis for DGE. This review first delineates gut microbial alterations in diabetes and mechanisms by which dysbiosis compromises the integrity of the ENS. Second, we analyze how microbiota-derived metabolites (short-chain fatty acids, bile acids, tryptophan), gut hormones (glucagon-like peptide-1, ghrelin), and neurotransmitters (acetylcholine, vasoactive intestinal peptide, nitric oxide) multitarget the ENS—collectively establishing the “microbiota-ENS axis” as the central hub for GI sensorimotor control. Finally, we provide an overview of preclinical and clinical evidence for microbiome-targeted therapies (probiotics, prebiotics, fecal microbiota transplantation) in alleviating DGE symptoms and repairing ENS while outlining translational challenges and future research priorities.

## 1 Introduction

Diabetes has become a global public health crisis, with epidemiological data revealing a prevalence of 10.5% among individuals aged 20–70 years, projected to climb to 12.2% by 2045 (Sun et al., 2021). Diabetic gastroenteropathy (DGE), a prevalent complication of diabetes, impacts the gastrointestinal (GI) tract from the esophagus to the colon, presenting clinical symptoms including esophageal motility disorders, gastroparesis, constipation, diarrhea, and fecal incontinence. In patients with diabetes mellitus, the incidence of GI symptoms is significantly higher than in those without diabetes, especially in women with poor glycemic control, long-standing diabetes, or other diabetic complications, although the reported prevalence of DGE varies widely by region (Du et al., 2018). Research indicates that 70% to 75% of diabetic individuals experience at least one GI symptom (Krishnasamy and Abell, 2018), with dysphagia (63%), gastroesophageal reflux (41%), early satiety or nausea (10%–20%), constipation (60%), and diarrhea (20%) being especially prominent (Concepción Zavaleta et al., 2021). These symptoms contribute to malnutrition, impaired drug absorption, decreased treatment adherence, and reduced quality of life. Despite the widespread use of validated tools, such as the Gastrointestinal Symptom Rating Scale (GSRS) and Gastroparesis Cardinal Symptom Index (GCSI), the diagnosis of DGE remains hindered by the lack of specific biomarkers and the reliance on exclusion criteria, resulting in high rates of underdiagnosis and clinical oversight (Revicki et al., 2003; Kulich et al., 2008). Nuclear gastric emptying scintigraphy is the gold standard for diagnosing gastroparesis, yet its practical use is limited due to radiation exposure and time-consuming procedures. Current treatments, such as prokinetics, antiemetics, and laxatives, aim to alleviate symptoms. Unfortunately, metoclopramide is the sole medication that has received Food and Drug Administration (FDA) approval for the treatment of gastroparesis. The utilization of domperidone, erythromycin, and mosapride (a 5-hydroxy tryptamine 4 agonist) is limited owing to safety concerns (Camilleri and Sanders, 2022). Novel approaches, including ghrelin receptor agonists, pyloric botulinum toxin injections, and surgical pyloromyotomy, show promise but necessitate thorough evaluation (Fleming et al., 2020; Schol et al., 2021). Therefore, clarifying the pathophysiology of DGE and discovering novel therapeutic targets are imperative.

Gastrointestinal function is coordinately regulated by two principal neural systems: the extrinsic autonomic nervous system and the intrinsic enteric nervous system. Prior investigations into the pathophysiology of DGE predominantly focused on autonomic dysfunction, particularly damage to the vagus nerve. Sustained hyperglycemia and its sequelae, including oxidative stress and inflammatory cascades, induce segmental demyelination and axonal degeneration in vagal fibers. These pathological changes disrupt bidirectional gut-brain communication and impair neuromodulation of gastrointestinal smooth muscle. However, contemporary research has progressively shifted toward elucidating impairment of the enteric nervous system (ENS). Termed the “second brain” of the gut, the ENS constitutes the largest autonomously functioning neural network within the GI tract. It orchestrates motility independent of the central nervous system (CNS) while maintaining anatomical and functional connectivity with the CNS via vagal pathways. First described by Albert von Haller in 1755 (Gershon, 1998), this system exhibits persistent GI motility even after intestinal disconnection from the brain. Analogous to the CNS, it integrates sensory neurons, interneurons, and motor neurons to form autonomous sensory-motor reflex arcs, which are the essential framework for the autonomous regulation of digestive tract functions (Niesler et al., 2021). Originating from vagal neural crest progenitors during embryogenesis, the ENS comprises millions of neurons and glial cells organized into myenteric (Auerbach’s) and submucosal (Meissner’s) plexuses. The myenteric plexus, spanning the GI tract, coordinates smooth muscle contractions to propel luminal contents, whereas the submucosal plexus, localized to the small and large intestines, modulates secretion, absorption, and responses to chemical and mechanical stimuli (Pawolski and Schmidt, 2020). Thus, key gastrointestinal functions including motility, sensation, and secretion are all regulated by the ENS, with its impairment contributing to a spectrum of gastrointestinal neuropathies. As observed in disease models of gastroparesis (Tseng et al., 2022), chronic constipation (Wang et al., 2023), functional dyspepsia (Tait and Sayuk, 2021), and irritable bowel syndrome (IBS) (Mayer et al., 2023), there is a notable loss of enteric neurons and ICCs, along with a reduction in the size and quantity of ganglia, underscoring the critical role of the ENS in maintaining GI function and motility. Nonetheless, the ENS, along with enteric neurons, enteric glial cells (EGCs), and ICCs, may be damaged from several diabetes-related variables, including chronic hyperglycemia, advanced glycation end products, oxidative stress, gut dysbiosis, and inflammation (Abdalla, 2024; Uppaluri et al., 2024). Undoubtedly, glycemic control remains the cornerstone for preventing and delaying the progression of DGE, while the search for targets to repair and regulate the ENS continues to be a major focus of current research.

Emerging evidence suggests bidirectional interactions between the ENS and gut microbiome (Sharkey and Mawe, 2023). The gut microbiome, the most complex microecosystem in the digestive tract, plays a pivotal role in various physiological processes, such as nutrient absorption, glucose and lipid metabolism, immune regulation, and GI motility (Del Chierico et al., 2022). Recent studies have demonstrated that the development, maturation, and integrity of the ENS are profoundly influenced by the gut microbiome. Research (Sajdel-Sulkowska, 2023) shows that the development of fetal ENS could be influenced by maternal gut dysbiosis during pregnancy. In adulthood, antibiotic-induced germ-free mice also exhibit a significant reduction in the number of enteric neurons, glial cells, and vagal afferent neurons, leading to extended small intestinal and prolonged GI transit time (Hung et al., 2020). However, these traits were reversible upon microbial colonization or supplementation with *Bacteroides* species or short-chain fatty acids (SCFAs), along with restoring cholinergic neuronal activity and promoting GI motility (Aktar et al., 2020). Thus, the gut microbiota may modulate the function and homeostasis of the ENS through its metabolites and signaling molecules. These findings suggest that microbiome-based therapies may represent an innovative approach to improve GI dysfunction (Simon et al., 2021). Nevertheless, despite these encouraging findings, existing research on the specific pathways by which gut microbiota modulate the ENS to ameliorate GI issues in patients with diabetes remains insufficient. There exists a notable gap in identifying viable treatment targets.

**Figure 1:**
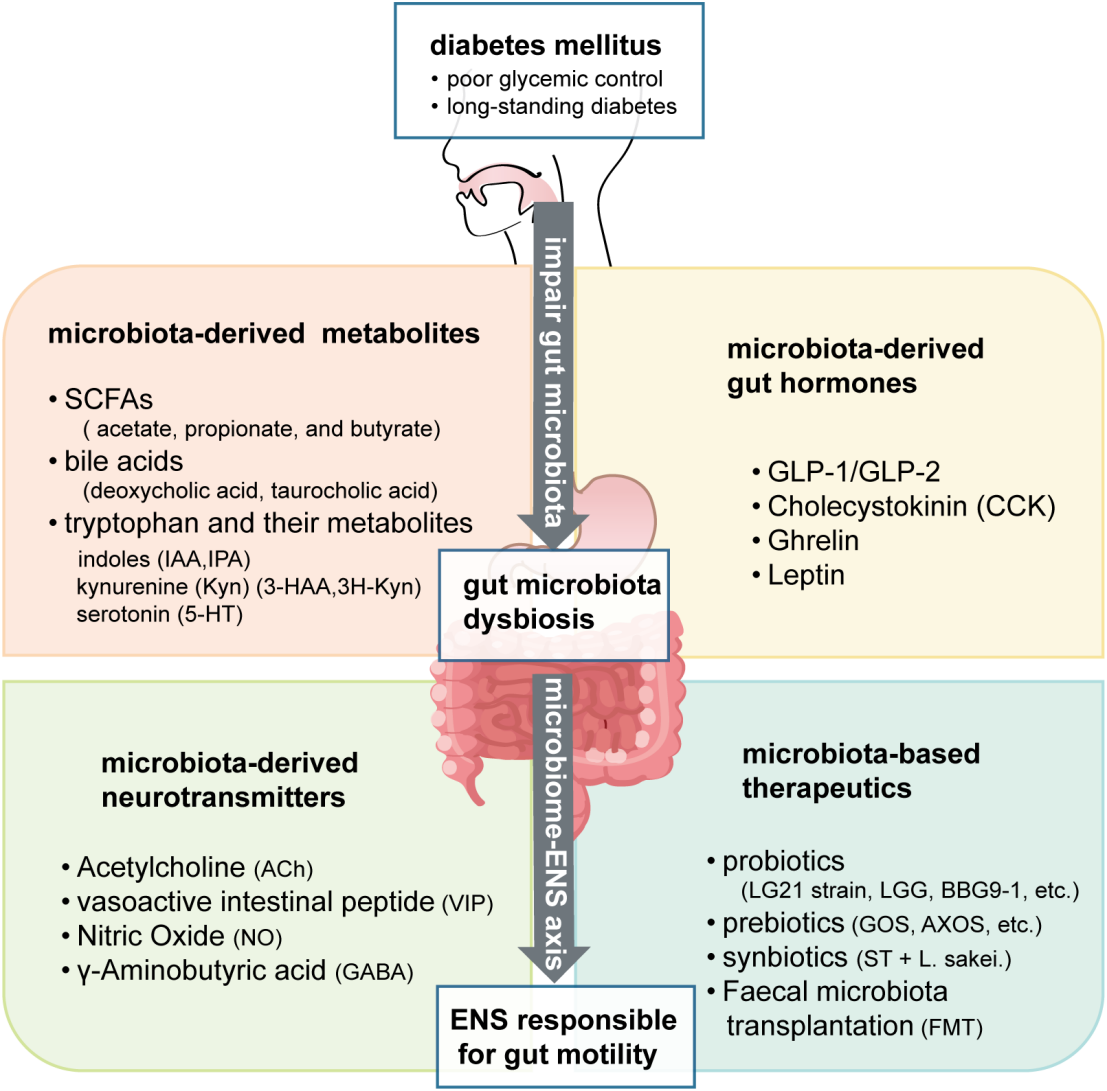
Mechanistic insights into diabetic gastroenteropathy: bidirectional interactions between diabetes and gut microbiota impacting the enteric nervous system.

This review aims to provide an update on the mechanisms and therapeutic potential of the “microbiome-ENS axis” in DGE, with a particular emphasis on the interactions between the gut microbiome and the ENS (**Figure 1**). We first elaborate on the impact of gut dysbiosis on the ENS and GI motility. Subsequently, we analyze the mechanisms by which microbiota-derived metabolites, gut hormones, and neurotransmitters regulate the ENS and GI function and provide a focused discussion on their contributions. Finally, we evaluate the translational potential of microbiome-based therapies, including probiotics, prebiotics, and fecal microbiota transplantation (FMT), to lay the theoretical groundwork for developing precision treatments of DGE and improve patients’ quality of life.

## 2 Mechanisms and consequences of diabetes-induced gut microbiota dysbiosis

### 2.1 Mechanisms underlying gut dysbiosis-induced diabetes

Although the gut microbiota is initially established at birth, it evolves dynamically throughout life, modulated by host age, dietary patterns, and physical activity (Rinninella et al., 2019). Dietary patterns differentially influence the pathogenesis of diabetes, potentially mediated by gut microbiota alterations. For instance, high-sugar or high-fat diets increase *Akkermansia*, *Proteobacteria*, and endotoxemia while elevating the *Firmicutes/Bacteroidetes* ratio and reducing *bifidobacteria* abundance (Sen et al., 2017). Protein sources differentially modulate the composition of gut microbiota: poultry and fish consumption increase *Actinobacteria*, beef consumption elevates *Bacteroidetes*, and soy protein enhances probiotics like *Lactobacillus* and *Bifidobacterium* and reduces *Bacteroides* (Wu et al., 2022a). These microbial shifts increase intestinal permeability, thereby promoting bacterial translocation and endotoxemia. Moreover, dysbiosis alters microbial metabolites, including short-chain fatty acids (SCFAs), trimethylamine N-oxide (TMAO), and indoles, which disrupts endocrine signaling (PYY, GLP-1/2, adiponectin, and resistin), impairs insulin pathways, and promotes lipogenesis, ultimately driving obesity and diabetes (Moszak et al., 2020). Conversely, meta-analyses demonstrate that high-fiber diets significantly increase the abundance of *Bifidobacterium* and *Lactobacillus* along with SCFAs, while suppressing enteropathogens (*Shigella, Escherichia coli, Klebsiella*) and improving glucose and lipids. Subgroup analyses reveal that specific prebiotics (fructans and galacto-oligosaccharides) further elevate the abundance of *Bifidobacterium* and *Lactobacillus* (So et al., 2018). Vegetable/fruit-rich diets reduce the risk of gestational diabetes by modulating *Lachnospiraceae, Blautia*, and *Ruminococcus* (Shan et al., 2024). Sedentary lifestyles, established obesity risk factors, may induce insulin resistance through altered Firmicutes/Bacteroidetes ratios (Sikalidis and Maykish, 2020). Regular exercise, conversely, promotes beneficial bacteria proliferation, enhances intestinal barrier integrity, and maintains metabolic homeostasis (Campaniello et al., 2022). Thus, dietary modification and physical activity represent pragmatic approaches for correcting metabolic dysregulation in diabetes. Interventions incorporating fiber, plant-based foods, probiotics, and prebiotics demonstrate efficacy in reversing metabolic syndrome through microbial remodeling (Upadhyaya and Banerjee, 2015).

### 2.2 Mechanisms underlying diabetes-driven microbiota remodeling

Notably, fecal microbiota transplantation (FMT) from obese type 2 diabetic donors into germ-free mice induces weight gain and glucose intolerance, underscoring the pivotal role of gut microbiota dysbiosis in the pathogenesis of diabetes (Pearson et al., 2022). However, the alterations of gut microbiota not only contribute to diabetes but are reciprocally reshaped by diabetes. As evidenced in db/db and ob/ob mouse models (Thaiss et al., 2018), sustained hyperglycemia activates intestinal epithelial glucose transporter 2 (GLUT2) and downregulates the expression of zonula occludens-1 (ZO-1), increasing intestinal permeability. This compromised barrier facilitates the translocation of harmful microorganisms, such as *Escherichia coli*, from the gut lumen into systemic circulation, thereby exacerbating systemic inflammation (Darra et al., 2023). Furthermore, significant reductions in butyrate producers (*Pseudoflavonifractor*, *Clostridium*, *Alistipes*, *Faecalibacterium*, *Oscillibacter*) and secondary bile acid producers (*Eubacterium rectale*, *Clostridium scindens*, *Bacteroides fragilis*) are observed in prediabetic and type 2 diabetic individuals. These microbial deficits disrupt intestinal barrier homeostasis, further promoting microbial translocation and perpetuating dysbiosis. Diabetes-associated chronic inflammation, characterized by elevated serum TNF-α and IL-6, further compromises gut immune equilibrium, amplifying barrier dysfunction and dysbiotic cascades.

### 2.3 Characteristic of diabetes-associated gut microbiota dysbiosis

A growing body of evidence (Wu et al., 2020; Zhao et al., 2020b; Zhou et al., 2022) reveals markedly altered abundance and diversity of gut microbiota in patients with diabetes compared to healthy individuals, characterized by low-diversity dysbiosis and an overgrowth of opportunistic pathogens. *Bacteroides* and *Bifidobacterium*, frequently implicated in type 2 diabetes, exhibit an inverse correlation with disease severity (Gurung et al., 2020). While an elevated *Firmicutes/Bacteroidetes* ratio is often associated with obesity (Koliada et al., 2017), numerous studies (Ahmad et al., 2023; Karačić et al., 2024) report non-significant or inverse correlations with obesity. Magne et al. (2020). attribute these discrepancies to methodological heterogeneity, including variations in sample size, participant characteristics, and sequencing approaches (16S rRNA vs. metagenomics). Notably, a systematic review (Zhou et al., 2020) indicated a decreased F/B ratio in fecal samples from type 1 diabetes (T1DM) mellitus patients analyzed via 16S rRNA sequencing, whereas duodenal biopsies revealed an increased ratio in Italian cohorts (Pellegrini et al., 2017, p. 1). This spatial heterogeneity underscores the necessity for segmental gut sampling (e.g., gastric mucosa, jejunal contents) coupled with spatial microbiome analysis to elucidate microbiota-host interactions.

Type 2 diabetes is associated with increased abundance of *Aspergillus, Micrococcus*, and *Actinomycetes*, alongside opportunistic pathogens (*Streptococcus, Clostridium, Escherichia-Shigella, Enterococcus, Klebsiella*) that contribute to metabolic endotoxemia (Zhou et al., 2022). Although reduction of *Akkermansia muciniphila* typically correlates with intestinal hyperpermeability, facilitating pathogen translocation and metabolic disease pathogenesis, paradoxical increases have been reported in some T2DM studies (Zhao et al., 2020b). This anomaly may arise from confounding factors such as metformin exposure or consumption of polyphenol-rich green tea, though mechanistic validation is pending (de la Cuesta-Zuluaga et al., 2017; Gurley et al., 2019). Such confounders necessitate stratified analyses, particularly given metformin’s documented stimulation of SCFA-producing taxa (*Butyrivibrio*, *Bifidobacterium bifidum*, *Megasphaera*, *Prevotella*, *Escherichia coli*), which may obscure true microbiota-disease relationships (de la Cuesta-Zuluaga et al., 2017).

## 3 Impacts of gut microbiota dysbiosis on gastrointestinal nerves and function

**Figure 2:**
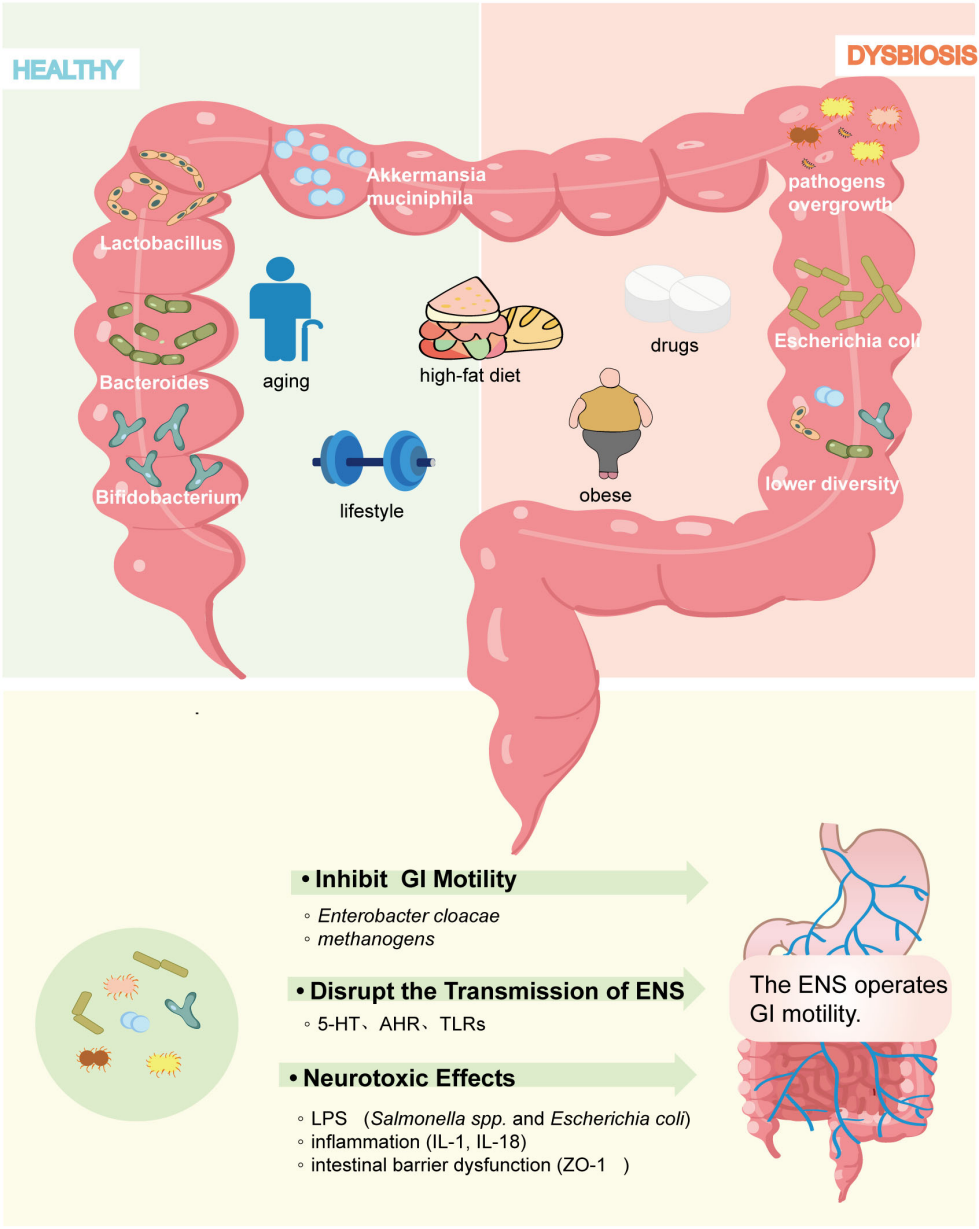
Factors influencing gut microbiome composition in diabetes models. Multiple variables, including age, dietary patterns, physical activity, hyperglycemia, obesity, and glucose-lowering agents, alter the abundance and composition of gut microbiota and contribute to diabetic gastrointestinal dysfunction.

In the diabetic state, gut microbiota dysbiosis further exacerbates GI motility dysfunction and impairs the ENS. Alterations in microbiota observed in both clinical and animal models of DGE underscore the association between these microbial communities and GI motility. Enrichment of *Proteobacteria* represents one of the most prominent microbiota features in DGE, with increased abundance positively correlating with the progression of DGE (Du et al., 2022). A small-scale Chinese clinical study (Lin et al., 2024) reported an increased abundance of *Proteobacteria, including Pseudomonas and Alkalibacterium*, in patients with DGE. A cohort study (Du et al., 2022) on diabetic autonomic neuropathy revealed a significant increase in taxa within *Proteobacteria*, including *Escherichia-Shigella, Escherichia coli*, and *Megasphaera*. *Actinobacteria, Proteobacteria*, and *Firmicutes* are dominant in DGE and show a significant positive correlation with impaired gastrointestinal motility (Huang et al., 2024; Zheng et al., 2024). Crucially, this diabetes-induced gut microbiota dysbiosis may profoundly adversely affect the ENS and impede GI motility, as evidenced by recent mechanistic investigations (**Figure 2**).

### 3.1 Inhibition of gastrointestinal motility

Studies indicate that GI motility can be directly impacted by the abundance of specific bacterial populations. For instance, an increase in certain specific microbiota has been found to be associated with reduced GI motility in patients with constipation, including potentially pathogenic bacteria such as *methanogenic bacteria*, *Desulfovibrionaceae*, *Escherichia coli*, and *Staphylococcus aureus* (Pan et al., 2022). A notable increase in the abundance of *Enterobacter cloacae* and *methanogens* is observed in patients with diabetes, which are the main sources of methane and hydrogen sulfide (Tian et al., 2022). It is reported that they are inversely related to colonic transit speed (Attaluri et al., 2010; Tang et al., 2018), and the mechanism involves the alteration of ion channel mechanisms. Clinical cohort studies (Feng and Li, 2022) have demonstrated that patients with diabetes have a 2.91-fold higher risk of developing small intestinal bacterial overgrowth (SIBO) compared to non-diabetic individuals. The delayed small intestinal transit time is closely associated with a positive lactulose breath test, also known as the methane-hydrogen test, further confirming that the overgrowth of hydrogen-producing and methanogenic bacteria can directly impede GI motility (Talamantes et al., 2024).

### 3.2 Disruption of the transmission of the ENS signaling

Research conducted by McVey Neufeld et al. (2015) revealed that germ-free mice exhibit lower excitability of intrinsic primary afferent neurons (IPANs) and mesenteric neurons in comparison to healthy controls. Although the precise mechanisms remain elusive, gut microbiota indirectly modulate the ENS signaling through multiple pathways, including interference with neurotransmitter biosynthesis and activation of aryl hydrocarbon receptors (AHR) and Toll-like receptors (TLRs). Serotonin (5-hydroxytryptamine, 5-HT), a critical neurotransmitter and paracrine signaling molecule, regulates diverse gastrointestinal functions by acting on neurons, smooth muscle cells, and immune cells (De Vadder et al., 2018). Beyond its production by enterochromaffin cells (ECCs), 5-HT is generated through tryptophan metabolism by *Bacteroides, Lactococcus, and Klebsiella* sp*ecies*, while its reuptake via the serotonin transporter (SERT) is inhibited by *Escherichia coli* (Esmaili et al., 2009). Although diminished 5-HT levels correlate with dysmotility in patients with diabetic gastroparesis, direct evidence linking microbiota dysbiosis to 5-HT reduction in this condition remains limited (Bharucha et al., 2019). Microbiota-derived 5-HT and its metabolites additionally serve as AHR agonists. Functioning as a ligand-dependent transcription factor, AHR bridges gut microbiota-ENS crosstalk by detecting microbial alterations that influence colonic motility development. Notably, both dysbiosis and 5-HT deficiency impair AHR-mediated ENS regulation (Obata et al., 2020). Additionally, TLRs recognize pathogen-associated molecular patterns and interfere with ENS signal transmission by detecting pathogen-associated molecular patterns (PAMPs) such as LPS and lipoproteins (Li and Wu, 2021). Anitha (Anitha et al., 2012), Yarandi (Yarandi et al., 2020), and their colleagues noticed that TLR2- and TLR4-deficient mice align with the mechanism responsible for gut dysmotility in germ-free mice, characterized by a diminished quantity of nitrergic inhibitory neurons. Marked decreases in glial cell line-derived neurotrophic factor (GDNF) were also observed in TLR2-deficient and microbiota-depleted mice, whereas supplementation with GDNF or TLR2 agonist restored the ENS function, indicating that both are prerequisites for ensuring the integrity of ENS (Brun et al., 2013).

### 3.3 Neurotoxic effects

Clinical studies (Gomes et al., 2017) indicate that serum lipopolysaccharide (LPS) levels in patients with T2DM are 1.66-fold higher than in healthy individuals. Hyperglycemia- and obesity-associated free fatty acid (FFA) and bile acid dysmetabolism selectively suppress SCFA-producing bacteria, including *Bifidobacterium*, *Faecalibacterium prausnitzii*, and *Roseburia*, while promoting opportunistic pathogens such as *Clostridium* and *Streptococcus* (Pinart et al., 2021). This dysbiosis compromises the synthesis of 5-HT and promotes endotoxemia. Furthermore, hyperglycemia induces GLUT2-dependent tight junction impairment, increasing intestinal permeability and establishing leaky gut (Di Vincenzo et al., 2024). This compromised barrier facilitates translocation of Escherichia coli and Salmonella, driving endotoxemia and low-grade chronic inflammation. Although low-dose LPS is essential for the survival of the ENS, chronic exposure to high-dose LPS triggers neurotoxicity and impairs viability through the activation of the TLR4/nuclear factor kappa-B (NF-κB) pathway (Anitha et al., 2012). Despite insufficient data demonstrating a direct correlation between the impaired ENS and higher LPS levels in diabetes models, studies on LPS-induced endotoxemia indicate that high doses of LPS lead to delayed gastric emptying and weakened contractile activity in the cecum and colon (De Winter and De Man, 2010). Beyond that, LPS activates NOD-like receptor thermal protein domain-associated protein 3 (NLRP3) inflammasomes in intestinal epithelial cells and stimulates dendritic cells and macrophages, leading to the release of various inflammatory factors, including interleukin-1β (IL-1β), IL-18, and TNF-α (Han et al., 2021; Chen et al., 2023). In mouse models, IL-1β (Lefèvre et al., 2023) and macrophage-derived (especially CD45+/CD11b+/F4/80+) inflammatory factors (Cipriani et al., 2018) have shown strong correlations with prolonged colonic transit and delayed gastric emptying. It also alters the structure and signaling of the ENS by inducing myenteric plexitis, neuronal hyperplasia, and neuropeptide dysregulation (Bubeck et al., 2023). Furthermore, LPS may downregulate ZO-1, leading to intestinal barrier impairment and exacerbating inflammatory responses (Wang et al., 2022c). Zogg et al. (2023). demonstrated that GI dysfunction can be ameliorated by restoring intestinal barrier integrity and mitigating inflammation-induced neurotoxicity. This research provides compelling evidence for the link between intestinal barrier impairment and gastrointestinal dysfunction. Collectively, these alterations disrupt GI motility via impaired ENS electrophysiology, neurotoxic damage, and dysregulated contractility. Nevertheless, future investigations must establish direct evidence linking specific microbial disruptions to ENS pathology in DGE models or clinical cohorts.

## 4 The gut microbiome-ENS axis in diabetic gastroenteropathy

**Figure 3:**
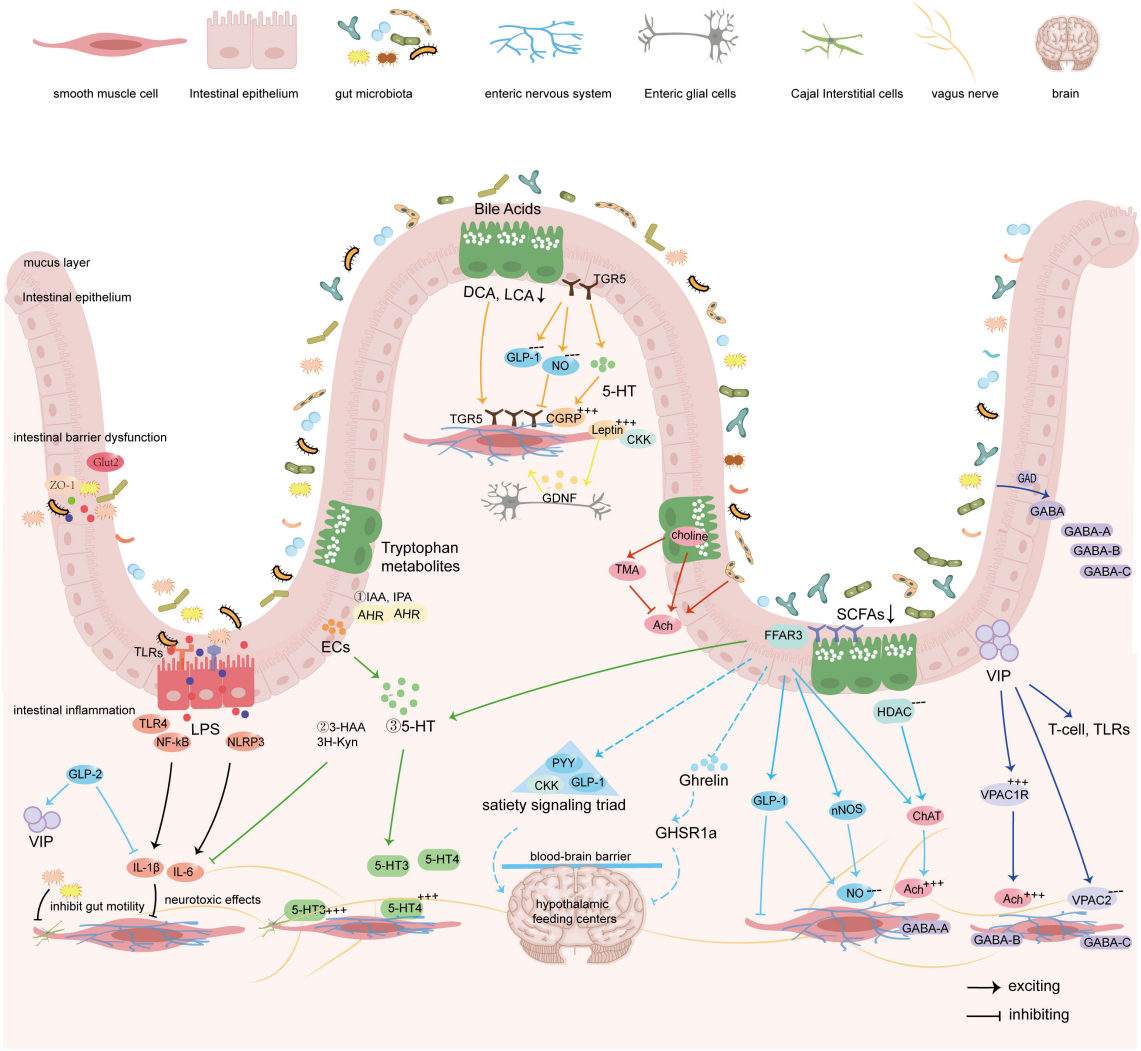
Gut microbiota regulates the sensation and motility of the gastrointestinal tract by modulating the ENS via its metabolites, gut hormones, and synthesized neurotransmitters.

Given the emerging evidence linking alterations in gut microbiota with ENS impairment, further exploration of these interactions could unveil novel strategies for mitigating the impact of diabetes on digestive health. In this section, we will focus on gut metabolites, as well as gut hormones and neurotransmitters regulated by the gut microbiota, elucidate their mechanisms of action in communicating with the ENS to influence diabetic gastrointestinal motility, while discussing the limitations of current research and potential future breakthroughs based on existing evidence. Given that distinct microbial derivatives act through unique molecular targets and pathways to regulate the enteric nervous system (ENS), this review adopts a categorical approach to elucidate the mechanisms underlying their crosstalk more clearly within the gut microbiota-ENS axis (**Figure 3**).

### 4.1 Microbiota-metabolites-ENS interactions

SCFAs, bile acids, and tryptophan along with its metabolites are primary metabolic products of the gut microbiota. In addition to providing energy to the host, these metabolites are essential for regulating GI physiological functions. This section provides an update on how these metabolites influence the ENS through complex signaling networks.

#### 4.1.1 Short-chain fatty acids

Short-chain fatty acids (SCFAs), the primary end products of anaerobic bacterial fermentation of dietary fibers in the mammalian colon, can reach concentrations of 50–200 mM, with acetate (C2), propionate (C3), and butyrate (C4) accounting for approximately 95% of total SCFAs (Louis and Flint, 2017). *Bacteroidetes* and *Firmicutes* serve as predominant producers of SCFAs: *Bacteroidetes* primarily generates acetate and propionate, whereas *Firmicutes* predominantly produces butyrate. Elevated butyrate associates with improved islet function, while increased propionate correlates with higher risk of type 2 diabetes (Sanna et al., 2019). Nevertheless, both butyrate and propionate modulate the ENS through three principal mechanisms:

Firstly, neurotransmitter modulation: SCFAs balance excitatory and inhibitory signals in the ENS by modulating certain neurotransmitters. As a potent histone deacetylase (HDAC) inhibitor, butyrate significantly increases the proportion of choline acetyltransferase (ChAT)-immunoreactive myenteric neurons, thereby enhancing cholinergic-mediated colonic circular muscle contractility (Soret et al., 2010). Concurrently, SCFAs activate enterochromaffin cells (ECCs) to upregulate tryptophan hydroxylase 1 (TPH1) transcription—the rate-limiting enzyme for 5-HT biosynthesis from tryptophan (Reigstad et al., 2015). 5-HT exerts its biological effects through specific receptors, including 5-HT1, 5-HT2, 5-HT3, 5-HT4, and 5-HT7, with SCFAs modulating both serotonin transporter (SERT) activity and receptor expression profiles (Buey et al., 2023). Notably, 5-HT4 receptor activation initiates cholinergic motor neuron-dependent circular muscle contraction. Critically, SCFAs activate free fatty acid receptor 3 (FFAR3/GPR41) on submucosal neurons, myenteric plexuses, and vagal ganglia, thereby remodeling GI motility reflexes through coordinated modification of nitrergic and cholinergic neurotransmission (Nøhr et al., 2015). Secondly, neuroprotective: SCFAs attenuate antibiotic-induced neuronal loss and regulate the survival of enteric neurons (Vicentini et al., 2021). Butyrate additionally improves GI motility and prevents ICC depletion in chronic constipation via AKT/NF-κB signaling (He et al., 2020). Thirdly, hormone regulation: Acting via vagal afferents or free fatty acid receptor 2/3 (FFAR2/FFAR3) activation on enteroendocrine cells, SCFAs stimulate secretion of glucagon-like peptide-1 (GLP-1) and peptide YY (PYY), suppressing appetite and delaying gastric emptying (Goswami et al., 2018).

Notably, SCFAs exert concentration-dependent effects on GI motility. Dass et al. (2007) demonstrated that low concentrations (1 mmol/L) exert negligible effects, while moderate-to-high levels (10–100 mmol/L) dose-dependently reduce peristaltic pressure thresholds, shorten contraction intervals, and diminish wave amplitudes, collectively impairing propulsive activity. Supratherapeutic concentrations (300 mmol/L) will disrupt intrinsic motility rhythms. Furthermore, a physiological SCFA concentration gradient exists along the gut: high cecal concentrations (>115 mmol/L) inhibit intestinal peristalsis, whereas lower concentrations in the distal colon, terminal ileum, and ileum (<10 mmol/L) primarily suppress longitudinal muscle high-frequency contractions via the ENS, promoting intestinal content propulsion (Ono et al., 2004). In DGE, hyperglycemia-induced dysbiosis likely disrupts this physiological concentration gradient, impairing ENS rhythmicity and precipitating dysmotility. Future investigations must establish optimal SCFAs therapeutic windows while developing strategies to mitigate high-concentration toxicity. Crucially, direct evidence demonstrating the efficacy of exogenous SCFA supplementation in restoring ENS function under diabetic conditions remains lacking, necessitating rigorous translational models to evaluate SCFA-mediated neuromodulation in DGE pathogenesis.

#### 4.1.2 Bile acid metabolism

Bile acids (BAs) serve as pivotal mediators in microbiota-host crosstalk, with their biotransformation from primary to secondary forms critically dependent on gut microbiota. Primary BAs are synthesized predominantly via hepatic 12α-hydroxylation pathways involving CYP7A1 and CYP8B1, while secondary BAs derive from non-12α-hydroxylated routes catalyzed by bacterial 7α-dehydroxylase. Given the absence of endogenous 7α-dehydroxylase in humans, conversion to secondary BAs depends on commensal bacteria, including *Clostridium* and *Bacteroides*, establishing gut microbiota as indispensable for BA metabolism (Jia et al., 2021). Secondary BAs, such as deoxycholic acid (DCA) and lithocholic acid (LCA), regulate GI motility by activating farnesoid X receptor (FXR) and Takeda G protein-coupled receptor 5 (TGR5). In diabetes, diminished abundance of *Akkermansia*, *Bacteroides*, *Bifidobacterium*, *Faecalibacterium*, and *Roseburia* reduces β-glucuronidase and 7α-dehydroxylase activities, thereby blocking the conversion of primary BAs to secondary BAs and consequently decreasing the synthesis of DCA and LCA (Li et al., 2021b). This microbiota-driven BAs dysmetabolism compromises intestinal barrier integrity and disrupts the ENS signaling via TGR5 receptors on ECCs and enteric neurons.

Notably, the widespread expression of TGR5 in enteric neurons, ECCs, and enteroendocrine cells (EECs) results in regional effects of BAs on GI motility. Studies (Ferrell and Chiang, 2019) have shown that intragastric administration of BAs impedes gastric emptying and small intestine transit through TGR5-mediated secretion of GLP-1. Bile acids also stimulate TGR5 on motor neurons, producing nitric oxide (NO) and inhibiting spontaneous contractions in the ileum (Poole et al., 2010). On the contrary, in the colon, DCA and taurocholic acid could activate TGR5 on ECs and enteric neurons to stimulate the release of TPH1 and 5-HT, thus enhancing colonic motility (Bunnett, 2014). The TGR5 receptor is a critical mediator of BAs in the GI tract. The deficiency of the TGR5 receptor results in constipation by delaying GI transit, reducing bowel movement frequency, and decreasing fecal water content (Zheng et al., 2022). Conversely, the overexpression of TGR5 results in diarrhea due to accelerating colonic transit in mice (Zhao et al., 2020a). Thus, given the region-specific effects of BAs and the concentration dependence of TGR5, future studies should further investigate how gut microbiota synergize with BAs and TGR5-mediated 5-HT release to regulate GI motility.

#### 4.1.3 Tryptophan and its metabolites

Tryptophan, an essential amino acid, exhibits intimate associations with gut microbiota functions through its metabolic pathways, particularly the synthesis of 5-HT that plays a pivotal role in regulating the ENS (Agus et al., 2018). Approximately 90% of intestinal 5-HT originates from ECCs, with gut microbiota directly modulating its synthesis and release via ECC interactions. Commensals, including *Lactococcus*, *Streptococcus*, *Escherichia coli*, and *Akkermansia muciniphila*, activate TPH1 in ECCs through SCFAs, catalyzing the conversion of tryptophan to 5-HT (Reigstad et al., 2015). And reduced abundance of *Bifidobacterium bifidum* similarly downregulates TPH1 and SERT expression, impairing 5-HT synthesis (Taverniti et al., 2021).

Within ENS signaling, 5-HT coordinates GI motility through ascending/descending interneuronal activation and vago-vagal reflexes by targeting multiple receptor targets (Keating and Spencer, 2019). Prokinetic receptors 5-HT3 and 5-HT4, densely localized to myenteric plexuses and autonomic terminals, mediate distinct effects: 5-HT4 activation evokes acetylcholine release from cholinergic neurons, enhancing smooth muscle contraction (Pauwelyn and Lefebvre, 2017), whereas 5-HT3 sensitization of afferent nerves regulates motility rhythms and secretory (Touhara et al., 2025). Experimental evidence (Israelyan et al., 2019; Spencer and Keating, 2022) confirms that mice lacking the 5-HT3 receptor exhibit slowed colorectal motility and prolonged gastrointestinal transit time due to a reduction in the number of enteric neurons, particularly dopaminergic and GABAergic neurons, underscoring the 5-HT–ENS axis’s necessity for GI motility. Paradoxically, microbiota-driven 5-HT excess induces hypermotility via 5-HT3 overactivation, underscoring the delicate equilibrium between microbiota and 5-HT.

Clinically, 5-HT3 receptor antagonists (e.g., alosetron) (Savarino et al., 2022) and 5-HT4 receptor agonists (e.g., prucalopride) (Barbara et al., 2023) treat diarrhea-predominant and constipation-predominant irritable bowel syndrome, respectively, reflecting differential microbiota effects on 5-HT signaling. In DGE, however, dysbiosis-induced cholinergic neuronal damage necessitates multimodal neuromodulatory strategies beyond single-receptor targeting. Future research should prioritize interventions coordinating 5-HT-producing microbiota with complementary pathways (like SCFAs or BAs metabolism) to achieve comprehensive repairment of the ENS.

### 4.2 Microbiota-hormones-ENS crosstalk

The GI tract functions as a significant endocrine organ, primarily due to specialized epithelial cells known as EECs. EECs, located in the mucosal lining from the stomach to the rectum, secrete over 20 hormones, including GLP-1, PYY, and cholecystokinin (CCK) (Adriaenssens et al., 2018). These hormones coordinate nutrient absorption, GI motility, and metabolic homeostasis through a dual mechanism: paracrine (acting on enteric neurons) and neuroendocrine (via vagal transmission) (Bany Bakar et al., 2023). Meanwhile, the release of gut hormones by EECs is influenced by gut microbiota and its metabolites, including SCFAs, bile acids, indoles, which changes GI homeostasis (Masse and Lu, 2023). In this section, we review several key hormones that play central roles in regulating the ENS, with a particular focus on GLP-1, CCK, ghrelin, and leptin.

#### 4.2.1 GLP-1 and GLP-2

As core components of the incretin system, glucagon-like peptide-1 (GLP-1) secreted by intestinal L-cells not only regulates glucose metabolism through glucose-dependent insulin secretion enhancement and glucagon suppression but also induces weight loss via gastric emptying delay and appetite inhibition. These dual actions establish GLP-1 receptor agonists (GLP-1RAs) as cornerstone therapies for type 2 diabetes and obesity (Nogueiras et al., 2023). Both GLP-1 and its homolog GLP-2 concentration-dependently enhance enteric neuronal survival, synergistically preserving the integrity of the ENS structure. GLP-2 further inhibits NF-κB signaling to reduce pro-inflammatory cytokines, such as TNF-α and IL-6, prevents inflammation-induced submucosal ganglion neuron loss, and increases vasoactive intestinal peptide-positive (VIP^+^) neuron proportions, collectively repairing neuroinflammatory damage (Abdalqadir and Adeli, 2022). However, the weight-loss-associated gastric emptying delay induced by GLP-1 remains contentious regarding potential exacerbation of diabetic gastroparesis and perioperative dysmotility (Camilleri and Lupianez-Merly, 2024). A recent systematic review (Hiramoto et al., 2024) indicates GLP-1RAs delay solid-phase gastric emptying by approximately 36 minutes, yet without statistically significant effects on liquid-phase emptying. Grasset et al. (2017). further demonstrated GLP-1-associated upregulation of nitric oxide synthase (NOS) expression in enteric neurons and vagal pathways. NOS catalyzes nitric oxide—an inhibitory neurotransmitter critical for ENS function. Nonetheless, the evidence remains insufficient to suggest that GLP-1RAs exacerbate GI motility; clinical vigilance and further high-quality studies are warranted.

Gut microbiota orchestrates GLP-1 secretion through metabolite-mediated mechanisms: firstly, SCFAs promote GLP-1 release via dual pathways: one is to activate the cell signaling pathway by binding to the FFAR2/FFAR3 receptor, which is impaired in FFAR2-deficient mice and shows decreased GLP-1 levels and glucose tolerance abnormalities (Tolhurst et al., 2012); the other is to increase the number of jejunal L-cells by inhibiting HDAC. Secondly, BAs synergistically enhance GLP-1 synthesis through TGR5/FXR co-activation on L-cells (Brighton et al., 2015). In type 2 diabetes, dysbiosis reduces SCFA, secondary BAs, and indole production, impairing jejunal L/K-cell activity and diminishing GLP-1/GIP secretion. This ultimately attenuates cholinergic neuron and vagal afferent activation efficiency within the ENS (Lee et al., 2012). Germ-free murine models (Yang et al., 2017) confirm that microbiota deprivation increases GLP-1R^+^ cells and prolongs gastrointestinal transit, whereas fecal microbiota transplantation normalizes GLP-1 secretion and motility, underscoring microbial indispensability in sustaining the GLP-1-ENS signaling axis.

#### 4.2.2 Cholecystokinin

CCK, a gut-brain peptide hormone predominantly secreted by duodenal and jejunal I-cells in response to lipids and proteins, is a key “satiety signaling triad” member alongside GLP-1 and PYY (Andermann and Lowell, 2017). CCK exhibits region-specific effects through its CCK1 receptor: In the gastric antrum and pylorus, CCK activates vago-vagal reflexes via CCK1 receptors, inhibiting antral contractions while enhancing pyloric sphincter tone to delay solid-phase gastric emptying, a mechanism validated by Lorenz and Goldman (Lorenz and Goldman, 1982). Direct antral smooth muscle hyperpolarization reduces action potential firing, further suppressing peristalsis. Within the gallbladder, CCK1 receptor stimulation induces smooth muscle contraction for bile expulsion. In the distal small intestine, CCK1 activation mobilizes intracellular calcium in smooth muscle cells and coordinates intersegmental peristalsis via vagal afferent fibers to facilitate chyme propulsion (Doong et al., 1998).

These CCK1-mediated effects remain unaltered by CCK2 receptor antagonists. Early CCK1 antagonists (e.g., loxiglumide) showed therapeutic potential for functional dyspepsia and gastroparesis (Chua et al., 1994), but clinical utility is limited by off-target effects, including abdominal pain and mood disturbances via action on anxiety-related brain regions and somatic nociceptive pathways (Li et al., 2024). Recent structural insights reveal that CCK1 receptor conformational plasticity enables selective Gs/Gi/Gq protein coupling, providing a molecular basis for developing gut-selective modulators (Zhang et al., 2021b). Gut microbiota regulates CCK secretion through interactions between metabolites and jejunal I-cells: SCFAs and bile acids enhance CCK synthesis by activating FFAR2 and TGR5 receptors on jejunal I-cells. Prebiotics such as inulin indirectly upregulate CCK expression by enriching SCFA-producing microbiota (Avirineni et al., 2022); however, whether SCFA-promoted CCK release in the stomach and intestines exacerbates GI motility disorders remains unclear.

#### 4.2.3 Leptin

Leptin, an adipocyte-derived endocrine hormone, regulates GI motility through binding leptin receptors (Ob-R) widely distributed on gastric vagal afferents and EECs. Notably, although leptin excites submucosal and myenteric neurons, it does not directly stimulate GI muscular activity but instead potentiates CCK-mediated intestinal propulsion (Reichardt et al., 2011). Mechanistically, leptin induces synthesis of glial cell line-derived neurotrophic factor (GDNF) within the ENS, preserving myenteric cholinergic neuron activity via GDNF-mediated neuroprotection. This effect counteracts high-fat diet-induced ENS damage in Western diet obesity models (Baudry et al., 2012). Leptin-deficient mice exhibit significantly delayed intestinal transit and barrier dysfunction, confirming its essential role in maintaining ENS structural and functional integrity (Tsai et al., 2020).

Gut microbiota modulates this process through leptin sensitivity regulation: probiotics predominantly comprising *Lactobacillus* and *Bifidobacterium* species significantly reduce serum leptin levels by mitigating endotoxemia and improving adipocyte secretory profiles (López-Moreno et al., 2020). In diabetic gastroenteropathy, dysbiosis-induced leptin resistance may exacerbate cholinergic neuronal injury and hypomotility by impairing the GDNF-ENS axis. As a negative regulator of insulin sensitivity, microbiota-dependent leptin modulation offers novel therapeutic targets for ameliorating diabetes-associated gastrointestinal symptoms.

#### 4.2.4 Ghrelin

Ghrelin, the sole orexigenic GI hormone, is produced by gastric X/A-like cells with secretion regulated by nutritional status, exhibiting fasting-induced elevation and postprandial suppression. As a pivotal modulator of ENS and metabolic homeostasis, ghrelin activates growth hormone secretagogue receptor 1a (GHSR1a) to exert dual regulatory effects (Rouault et al., 2020). Centrally, it stimulates neuropeptide Y (NPY) neurons and vagal afferents to coordinate feeding behavior and glucose metabolism (Nunez-Salces et al., 2021). Peripherally, ghrelin enhances cholinergic neuron density and activity in gastric myenteric plexuses and vagal pathways, augmenting contraction frequency and amplitude to accelerate solid-phase gastric emptying. This central-peripheral synergy underscores its therapeutic potential for DGE. Phase 2B study demonstrates that the ghrelin receptor agonist relamorelin shortens gastric emptying time in patients with DGE and alleviates vomiting via central antiemetic effects (Camilleri et al., 2017). The novel agonist HM01 increases myenteric cholinergic neurons by 50%, effectively ameliorating dysmotility associated with abdominal surgery or Parkinson’s disease (Yuan et al., 2023).

However, clinical translation is limited by metabolic sequelae: Ghrelin inhibits pancreatic β-cell KATP channels to reduce insulin secretion, potentially exacerbating postprandial glycemia—a critical concern in DGE. A primary underlying cause is the disruption of the ghrelin signaling axis by gut microbiota dysbiosis: in diabetic states, an elevated *Firmicutes/Bacteroidetes* ratio will induce ghrelin resistance (Ahmed et al., 2024), and specific bacterial groups (e.g., *Clostridium, Ruminococcus*) show positive correlations with ghrelin levels, whereas *Bacteroides* and *Bifidobacterium* show bidirectional relationships contingent on host metabolism (Leeuwendaal et al., 2021). Microbial metabolites also participate in regulating ghrelin levels through multiple mechanisms: LPS and the gaseous signaling molecule hydrogen sulfide (H^2^S) can interfere with intracellular ghrelin signaling (Slade et al., 2018); SCFAs act in a concentration-dependent manner, with low concentrations inhibiting ghrelin secretion by antagonizing GHSR1a, whereas excessive acetate activates the parasympathetic nervous system to promote its release. Therefore, future studies should focus on the precise regulation of the ghrelin-ENS pathway by specific functional microbiota, providing a basis for developing DGE treatment strategies that balance motility improvement and glycemic stability.

### 4.3 Microbiota-neurotransmitters-ENS pathways

Emerging evidence suggests that the gut microbiota plays a key role in neurological disorders by directly synthesizing neurotransmitters and regulating host neurotransmitter metabolism. It has been proved that gut microbiota possesses genes that encode neurotransmitter-synthesizing enzymes like glutamate decarboxylase and tryptophan hydroxylase (Banerjee et al., 2021; Otaru et al., 2021). Furthermore, microbial metabolites such as SCFAs could regulate neurotransmitter production in enteric neurons through epigenetic modifications (He et al., 2024b). Neurotransmitters such as acetylcholine (ACh), 5-HT, γ-aminobutyric acid (GABA), and nitric oxide (NO) are pivotal in regulating the ENS and GI motility. In this section, we systematically analyze the microbiome-neurotransmitter interaction network and explore its potential as a therapeutic target for DGE.

#### 4.3.1 Acetylcholine

Acetylcholine (ACh), the primary excitatory neurotransmitter of ENS cholinergic neurons, is synthesized by choline acetyltransferase (ChAT) and modulates GI functions through muscarinic receptors (M2/M3). M3 receptors mediate smooth muscle contraction and glandular secretion, predominantly driving motility, while M2 receptors indirectly regulate tone via inhibitory G-protein pathways (Foong et al., 2015). Gut microbiota orchestrates ACh homeostasis through multifactorial mechanisms: specific strains (e.g., *Lactobacillus plantarum Lsi*) secrete ACh-like compounds, directly enhancing intestinal contractions (Fujita et al., 2024); conversely, trimethylamine (TMA)-producing *Firmicutes* and *Proteobacteria* metabolize dietary choline, depleting ACh synthesis substrates (Eslami et al., 2024). In diabetic mice models, this metabolic shift correlates with downregulated expression of ACh receptors, kinases, and substrate, potentially impairing ENS cholinergic signaling (Tanase et al., 2020; Xu et al., 2020).

Microbiota-driven ACh dysregulation is validated in inflammatory bowel disease (IBD) models: Dysbiosis-induced choline deficiency is reversed by cytidine diphosphate (CDP)-choline supplementation, which upregulates choline transporter (ChT1), acetylcholinesterase (AChE), and α7 nicotinic acetylcholine receptor (α7nAChR) expression to restore neurotransmission (Guo et al., 2023a). In primary dysmotility or diabetic autonomic neuropathy, nAChR autoantibodies block ganglionic transmission, exacerbating dysmotility (Vernino et al., 2000). Butyrate counteracts this by inhibiting HDAC to promote regulatory T-cell (Treg) differentiation, reducing autoantibody production, and indirectly preserving cholinergic signaling (He et al., 2024a). Furthermore, SCFAs activate FFAR2/FFAR3 receptors to directly release cecal ACh, further reinforcing excitatory drive within the ENS (Ballout et al., 2021). Collectively, these findings delineate the core role of the “microbiota-SCFA-ACh” axis in sustaining ENS cholinergic activity.

#### 4.3.2 Vasoactive intestinal peptide

Vasoactive intestinal peptide (VIP), predominantly secreted by neurons in the myenteric and submucosal plexuses of the ENS, serves as a pivotal neurotransmitter coordinating gastrointestinal functions through dual VPAC1/VPAC2 receptor signaling. VPAC2 receptors enriched in gastrointestinal smooth muscle mediate VIP-induced relaxation of gastric fundus circular muscle (Robberecht et al., 1998). Conversely, VPAC1 receptors highly expressed in colonic mucosa regulate epithelial ion transport, mucus secretion, and barrier integrity, while their co-localization with ChAT on enteric neurons facilitates acetylcholine release to enhance longitudinal muscle contraction (Fung et al., 2014). Beyond neuromodulation, VIP maintains intestinal immune homeostasis by regulating T-cell responses and TLR signaling, demonstrating therapeutic potential in neuroimmune disorders like T1DM and irritable bowel syndrome (Iwasaki et al., 2019).

VIP is critical for preserving ENS function: VIP-deficient mice exhibit attenuated jejunal motility (Lelievre et al., 2007), and downregulated VIP expression correlates with severe constipation, indicating attenuated VIP signaling as a common pathological feature in dysmotility (Bai et al., 2023). Gut microbiota regulates VIP with striking strain specificity: VIP gene expression is significantly reduced in germ-free animal models, while colonization with *Escherichia coli* (but not *Lactobacillus*) restores its levels, suggesting specific microbial metabolites modulate VIP synthesis (Bai et al., 2023). Nevertheless, modulation of VIP activity by gut microbiota remains relatively limited. How gut dysbiosis in diabetes affects VIP activity remains unclear, as does whether this activity can be altered by regulating microbial species and abundance. Future studies should prioritize elucidating microbiota-VIP crosstalk in ENS regulation to advance microbiome-based therapeutics for GI motility disorders.

#### 4.3.3 Nitric oxide

Nitric oxide (NO), a key inhibitory neurotransmitter, is synthesized by NOS via the catalysis of L-arginine. Among its three isoforms (endothelial eNOS, neuronal nNOS, and inducible iNOS), nNOS-positive neurons predominantly regulate smooth muscle tone via the NO-cGMP pathway, mediating relaxation of the lower esophageal sphincter, pyloric sphincter, and Oddi sphincter, as well as coordinating gastrointestinal peristaltic rhythms (Groneberg et al., 2016; Idrizaj et al., 2021). In patients with diabetic gastroparesis, typical features include apoptosis of nitrergic neurons, downregulated nNOS and NO levels, accompanied by disrupted connections between nNOS^+^ neurons and SIP syncytia, leading to rhythmic disturbances in the jejunum, ileum, and colon, which is closely linked to gut microbiota dysbiosis (Bódi et al., 2019; Sanders and Ward, 2019; Camilleri and Sanders, 2022). Gut microbiota influence NO-mediated ENS effects through both direct synthesis and indirect regulation: certain *Lactobacillus* strains (e.g., *Lactobacillus lactis*, *Lactobacillus plantarum*) can directly secrete NO to induce ileal smooth muscle relaxation (Yarullina et al., 2016). Further evidence from antibiotic intervention experiments (Yarandi et al., 2020) shows that ampicillin treatment, which reduces Gram-positive bacteria and enriches Gram-negative bacteria, significantly decreases colonic nNOS enzymatic activity and nitrergic neuron count in mice, impairing colonic motility; notably, microbial recovery after antibiotic withdrawal reverses this phenotype, highlighting the critical role of microbiota structural stability in maintaining the NO-ENS axis.

#### 4.3.4 Gamma-aminobutyric acid

Gamma-aminobutyric acid (GABA), a dual-function neurotransmitter with both excitatory and inhibitory properties in the ENS, regulates gastrointestinal functions via ionotropic (GABA_A/C) and metabotropic (GABA_B) receptors, exhibiting segment-specific and receptor subtype-specific effects: in the stomach, GABA_A receptor activation mediates smooth muscle relaxation through NO release, while GABA_B receptors promote contraction by enhancing cholinergic signaling; in the duodenum, GABA_A receptors are involved in releasing ACh, and GABA_C receptors are associated with releasing NO (Tonini et al., 1989; Zizzo et al., 2007; Rotondo et al., 2010; Auteri et al., 2014, 2015). This regional specificity arises from differences in receptor distribution between ENS neurons and smooth muscle cells, which will significantly limit the clinical translation of effects targeting DGE. Gut microbiota represents an important source of GABA; genera such as *Bacteroides*, *Lactobacillus*, and *Levilactobacillus brevis* directly secrete GABA by encoding glutamate decarboxylase (GAD), a key enzyme in GABA synthesis (Banerjee et al., 2021; Otaru et al., 2021). In diabetic states, reduced abundance of these genera leads to weakened GABAergic signaling, impairing ENS regulation of intestinal motility and mucosal secretion (Strandwitz et al., 2019; Konstanti et al., 2024). However, the causal relationship between decreased GABA levels and gut dysbiosis in diabetes remains unclear: whether microbiota dysbiosis causes reduced GABA or hyperglycemia directly inhibits GAD activity requires further experimental verification.

## 5 Therapeutic strategies targeting the gut microbiome-ENS axis

Gut microbiota and their metabolites influence the ENS and GI function through multiple pathways and targets. Leveraging this advantage to develop systematic therapeutic approaches could represent a breakthrough in addressing DGE. At the 2011 annual meeting of the International Scientific Association for Probiotics and Prebiotics (ISAPP), experts reviewed the role of these microbial agents in neurogastroenterology, providing evidence for the interaction between the microbiome and the ENS (Saulnier et al., 2013). Probiotics, prebiotics, and synbiotics have been shown in extensive clinical studies (Markowiak and Śliżewska, 2017; Quigley, 2019; Mokkala et al., 2021; Wang et al., 2022a) to effectively treat gastrointestinal disorders, like functional dyspepsia, IBS, and chronic constipation, as well as metabolic diseases such as obesity, insulin resistance, and T2DM. Based on the “microbiome-ENS axis,” we therefore provide a summary of the preclinical and clinical data on the role of microbiota in regulating the ENS and improving GI motility.

### 5.1 Probiotics, prebiotics, and synbiotics

Probiotics, prebiotics, and synbiotics, as core interventions targeting the gut microbiota-ENS axis, exhibit tremendous potential in repairing neural damage and improving motility disorders in DGE by reshaping microbial structure and metabolic profiles (as detailed in **Tables 1**, **2**). As live non-pathogenic microorganisms that confer beneficial effects when consumed in adequate amounts, probiotics primarily include genera such as *Lactobacillus, Bifidobacterium, Bacillus*, and *Saccharomyces*, which exist in forms like fermented foods and enteric-coated capsules (Liu et al., 2018). Their core mechanism involves regulating the composition of gut microbiota to increase concentrations of SCFAs and secondary BAs, thereby activating ECCs to release 5-HT, enhancing ACh secretion from cholinergic neurons, and optimizing ENS inhibitory neural signals via the neuronal nNOS pathway, ultimately promoting gastric emptying and regulating intestinal transit rhythms (Huang et al., 2023; Liu et al., 2023). Prebiotics, as indigestible dietary fibers that resist direct digestion by the host gastrointestinal tract but selectively promote the proliferation of beneficial bacteria, such as inulin, fructooligosaccharides (FOS), galactooligosaccharides (GOS), lactulose, and resistant starch (Hutkins et al., 2016), indirectly enhance the function of the ENS by enriching SCFA-producing microbiota while synergistically regulating gastrointestinal hormone release to accelerate GI motility (Lee et al., 2024). Synbiotics further amplify these effects through the synergistic action of probiotics and prebiotics; for example, stachyose combined with *Latilactobacillus sakei* repairs ICC networks by increasing 5-HT and substance P (SP) levels, inhibiting expression of VIP and NOS, with significantly greater efficacy in promoting GI peristalsis than single-agent interventions (Guo et al., 2023b).

**Table 1:** Evidence of probiotics in gastroenteropathy models.

Types of probiotics		Subjects	Intervention duration	Key findings	Advantages and limitations	Reference
Lacticaseibacillus	Lactobacillus gasseri OLL2716 (LG21 strain)	Participants aged 20–64 years with mild to moderate delayed gastric emptying	For 12 weeks	Reduced salivary amylase concentration. Increased parasympathetic nervous activity. Enhanced gastric emptying capacity with a 4.1-fold greater than placebo.	1. A double-blind randomized controlled trial, high evidence level. 2. Favorable safety profile; no specific adverse events. 3. Small sample size (n=28). 4. Significant gender imbalance; female predominance.	(Ohtsu et al., 2021)
	LimosiLactobacillus pentosus CQZC02	BALB/c female mice (4 weeks) with loperamide-induced constipation	For 8 days	Accelerated gastric motility and colon transit time. Elevated serum levels of gastrin (Gas), MTL, and SP, along with reduced levels of endothelin-1 (ET-1), SS, and VIP. Increased expression of nNOS and iNOS neuronal in small intestinal tissues.	1. Conducted only in female mice; limited reproducibility. 2. Single model: loperamide-induced constipation model.	(Liu et al., 2023)
	JY: Lacticaseibacillus paracasei JY062 JM: Lactobacillus gasseri	KM male mice (6–7 weeks) with loperamide	For 2 weeks	The MIX group outperformed the others in promoting intestinal peristalsis and gastric emptying. Increased MTL and GAS levels, and decreased PYY levels. Increased 5-HT and NO levels, but no difference in VIP levels. Decreased apoptosis of ICC cells. Increased the abundance of beneficial bacteria (*Lactobacillus, Rikenellaceae* and *Clostridiaceae_Clostridium*) and the concentration of SCFAs.	1. Single model: loperamide-induced intestinal dysmotility with minimal effect on gastric motility.	(Cheng et al., 2023)
	Lactobacillus plantarum TWK10 (TWK10)	Sprague–Dawley male rats (6 weeks) with high-fat diet (HFD) and loperamide	For 5 weeks	High-fat-fed rats induced obesity, constipation, and slowed gastrointestinal transit time. TWK10 intervention increased fecal moisture content and intestinal transit rate. Decreased serum SS and CGRP levels, while increasing serum Ache levels. Enhanced the abundance of SCFA.	1. High-fat diet combined with loperamide-induced obesity-constipation model, closer to gastrointestinal dysfunction model induced by metabolic disorders. 2. Lacks clinical trial evidence.	(Liu et al., 2025)
	Lactobacillus rhamnosus GG (LGG)	C57BL/6 male mice (8–12 weeks)	For 2 weeks	Increased bowel frequency and reduced intestinal transit time. Modulated the ENS via formyl peptide receptor 1 (FPR1) and redox pathways. Enhanced expression of ChAT and SERT.	1. Elucidated mechanism of LGG improving gastrointestinal motility. 2. Lacks clinical evidence.	(Chandrasekharan et al., 2019)
Bifidobacterium	Bifidobacterium bifidum G9-1 (BBG9-1)	Patients on metformin with diabetic gastrointestinal symptoms (n=40)	For 10 weeks	Improved the GSRS total score. Increased *Firmicutes*/*Bacteroidetes ratio*. Decreased the abundance of the genus *Sutterella*.	1. An open‐label single‐arm exploratory. 2. Favorable safety profile; no serious adverse events. 3. Small sample size; GSRS scores relatively subjective.	(Hata et al., 2022)
	Bifidobacterium bifidum YIT 10347	patients with functional gastrointestinal disorders (n=100)	For 4 weeks	1. Improved gastrointestinal symptoms, flatulence, and diarrhea. 2. Improved frequency scale for symptoms of GERD (m-FSSG) scores; significantly improved when Gastrointestinal Symptom Rating Scale (GSRS) scores were below the median. 3. Failed to improve gastric emptying rate.	1. A double-blind, randomized, placebo-controlled study; 2. Adverse events like constipation may occur. 3. Controversy exists regarding improvement of upper and lower gastrointestinal symptoms.	(Gomi et al., 2018)
	Bifidobacterium bifidum CCFM1359	C57BL/6 male mice (8 weeks) with intestinal dysfunction	For 2 weeks	Alleviated symptoms. Increased the abundance of *Bifidobacterium* and valeric acid, and decreased *Enterococcus faecalis.* Upregulated expression of ACh while downregulating expression of nNOS to remodel the ENS.	1. Specific model: Animal model of intestinal dysfunction induced by senna extract, characterized by BDNF downregulation. 2. Limited to studying the role of neurotrophic factors.	(Wang et al., 2025)
	Bifidobacterium bifidum CCFM1163	C57BL/6J male mice (8 weeks) with cathartic colon	For 2 weeks	Reduced intestinal transit times. Increased abundance of *Bacteroides* and decreased abundance of *Proteobacteria.* Enhanced mRNA expression of ZO-1, Occluding, and Claudin-1 in the colon, contributing to intestinal barrier dysfunction. Decreased expression of TNF-α, IL-1β, and IL-6. Increased gene expression of TPH1 and 5-HT levels in the colon, aiding in the repair of the ENS.	1. Elucidated the link between gut microbiota and ENS. 2. By comparing efficacy among Bifidobacterium bifidum CCFM1163, Bifidobacterium M3, and Bifidobacterium bifidum M7, revealed strain-specific differences in efficacy.	(Tang et al., 2023)
	Bifidobacterium animalis subsp. lactis HN019	Adults aged 18–70 years with functional constipation (n=228)	For 4 weeks	1. Abdominal X-ray assessment of colonic transit time: no significant difference between groups. 2. Subgroup analysis: increased defecation frequency in patients with ≤3 bowel movements/week 3. No significant improvement in bloating, abdominal pain, straining during defecation, or stool consistency.	1. A double-blind, randomized, placebo-controlled, and dose-ranging trial. 2. Adequate sample size; high evidence level.	(Ibarra et al., 2018)
Lacticaseibacillus +Bifidobacterium	Probiotic group: Bifidobacterium animalis subsp. lactis HN019 + Lacticaseibacillus rhamnosus HN001	Adults aged 18–70 years with functional constipation (n=250)	For 4 weeks	Increased bowel movement frequency and reduced degree of defecation straining in all groups. Decreased plasma 5-HT levels in the probiotic group. Increased *Bifidobacteria* and SCFAs in the prebiotic group.	1. A double-blinded randomized placebo trial, adequate sample size; high evidence level. 2. Wide age range of participants. 3. Lacks microbial metabolites data.	(Lai et al., 2023)
Bacillus	Weizmannia coagulans BC99	Adults over 20 years of age with chronic constipation (n=90)	For 8 weeks	Improved constipation symptoms and quality of life. Increased levels of 5-HT, MTL, Ach and BDNF. Increased levels of anti-inflammatory factors (IL-4, IL-10) and decreased levels of pro-inflammatory factors (IL-6, IFN-γ). Altered the abundance of 93 metabolites.	1. A randomized, double-blind, placebo-controlled trial, high evidence level. 2. Incorporated serum metabolomics; lacks research on gut microbiota and SCFAs.	(Fan et al., 2025)
	Spore-forming Bacillus coagulans SNZ 1969 (BC)	Adults over 20 years of age with chronic constipation (n=80)	For 8 weeks	1. BC-treated group: significantly reduced total colonic transit time and increased defecation frequency 2. Increased Lactobacillales; decreased Synergistales. 3. Lactobacillales and Synergistales negatively and positively correlated with colonic transit time, respectively	1. A randomized, double-blind trial with a 2-week run-in period, high evidence level 2. Lacks exploration of associations between serum metabolomics and gut microbiota.	(Kang et al., 2021)
Escherichia	Escherichia coli Nissle 1917	C57BL/6J male mice (6 weeks) with constipation	For 2 weeks	Increased relative fecal water content and frequency of fecal defecation and shortened whole-gut transit time. Increased 5-HT levels and 5-HT4 receptors in colon. Performed anxiolytic effects and ameliorated depression-like behaviors. Increased microbiota diversity and abundance of *Alistipes*.	1. Demonstrated that E. coli-derived 5-HT significantly enhances intestinal motility; compared with 5-HT4 agonist prucalopride, which disrupts microbiota homeostasis, it also alleviates constipation-induced depression and anxiety.	(Li et al., 2022)
Saccharomyces	Saccharomyces boulardii CNCM I-745	C57BL/6J male mice (6 weeks) infected with HSV-1	For 4 weeks	Improved HSV-1 induced GI dysfunction. Repaired ileal neuromuscular structures. Restored the structure of the ENS, decreased nNOS levels and increased SP levels. Decreased levels of IL-4 and IL-10 in the intestine.	1. HSV-1-induced intestinal dysmotility model; provides reference for animal models. 2. Animal model relatively singular; difficult to fully simulate disease states.	(Brun et al., 2017)
Pediococcus	Pediococcus pentosaceus Li05 (Li05)	Male Wistar rats (4 weeks old) with IBS-D	For 3 weeks	1. Reduced mucosal and systemic inflammatory factors such as IL-1β, IL-6, and TNF-α. 2. Decreased ECs counts and serum 5-HT levels. 3. Reduced the abundance of harmful bacteria associated with inflammation including *Dubosiella* and *Erysipelatoclostridium* and increased beneficial bacteria including *Alloprevotella*, *Anaerotruncus*, and *Mucispirillum.* 4. Gut microbiota influence metabolites including linoleic acid, L-cysteine, and L-threonine.	1. Correlation analysis revealed probiotics improve gastrointestinal symptoms by regulating gut microbiota and affecting metabolomics; however, correlation analysis is insufficient to confirm mechanisms, requiring further experimental research.	(Wu et al., 2024)

**Table 2:** Evidence of prebiotics in gastroenteropathy models.

Types of prebiotics	Subjects	Intervention duration	Key findings	Advantages and limitations	Reference
BiomeBliss^®^ powder (including inulin, blueberry extract, β-glucan (oats), soy protein isolate, pomegranate flavor, xanthan gum, citric acid, stevia extract)	Youth-onset T2DM on metformin (n=6)	Phase 1: A 5-week period involved two groups receiving metformin alone or in conjunction with prebiotic supplements. Phase 2: A subsequent 4-week phase where in all participants consumed both metformin and prebiotic supplements.	Altered β-diversity of the microbiota with prebiotic supplements. Increased enrichment of SCFA-producing bacteria, such as *Bifidobacterium adolescentis, Blautia, and Acinetobacter.* Reduced the abundance of other bacteria (such as *Firmicutes* and *Roseburia* spp.). There was no significant difference in improving GI symptom scores or stool frequency.	1. Preliminary randomized controlled trial. 2. Small sample size. 3. Insufficient to demonstrate prebiotic efficacy in improving gastrointestinal symptoms.	(Dixon et al., 2023)
A prebiotic supplement (2.8 g/d Bimuno containing 1.37 g β-GOS)	patients with functional gut disorders (n=44)	For 4 weeks	Reduced the number of daytime anal gas evacuations, despite no significant improvement in bloating or bowel sounds. Increased the abundance of *Bifidobacterium* and reduced the abundance of *Bilophila wadsworthia*.	1. A randomized, two-centre, parallel, and double-blind study. 2. Inadequate sample size.	(Huaman et al., 2018)
The wheat bran extract Arabinoxylan-Oligosaccharide (AXOS)	Patients aged 20–55 years with whole-gut transit time (WGTT) >35 h determined by radio-opaque marker method (n=48)	For 12 weeks	Softened stool consistency without a significant alteration in WGTT. Reduced serum GLP-1 levels; yet no significant differences were detected in in PYY, insulin levels, hunger, or satiety scores. Enhanced fecal *Bifidobacterium* population; decreased microbial diversity, yet no significant changes in SCFAs concentration.	1. A double-blind, randomized, placebo-controlled, parallel trial; high evidence level. 2. Included healthy participants rather than obese or T2DM individuals.	(Müller et al., 2020)
Galactooligosaccharide (GOS)	Patients aged 19–75 years with functional constipation (n=63)	For 4 weeks	Increased bowel movement frequency and stool moisture and accelerated colonic transit time. Reduced *Firmicutes*/*Bacteroidetes* ratio. Increased the abundance of *Bifidobacterium* and *Lactobacillus* at the genus level.	1. A randomized, double-blind clinical trial; relatively high evidence level. 2. Primary endpoints: defecation frequency, stool consistency, and quality of life questionnaires; highly subjective.	(Lee et al., 2024)
pasta enriched along with the prebiotic inulin	healthy young male volunteers(n=20)	A 2-week run-in period and two 5-week study periods (11% inulin-enriched/control pasta), with an 8-week wash-out period in between.	1. Increased neurotensin (NT) and somatostatin (SS) 2. Delayed gastric emptying time 3. Reduced triglyceride and glucose level.	1. A randomized, double-blind crossover trial. 2. Small sample size; insufficient reproducibility.	(Russo et al., 2011)
prebiotic sesame sugar (PSC) (isomalto-oligosaccharide, konjac glucomannan and sesame)	C57BL/6 male mice (6 weeks) with constipation	For 2 weeks	Accelerated the small intestinal transit time. Increased the content of short-chain fatty acids in feces. Increased serum MTL and SP levels, reduced serum NO and SS levels.	1. Preliminary mechanistic exploration. 2. Lacks reverse validation.	(Xia et al., 2022)
Prebiotic: Wheat bran arabinoxylan (WBAX) Symbiotic: WBAX + *Lactobacillus reuteri* (*L. reuteri)* Postbiotic: pasteurized *L. reuteri* cultured with WBAX	Fecal bacteria from patients with T2DM and male C57BL/6J mice (6 weeks) with Colitis	For 4 weeks	Promoted microbial conversion of tryptophan (indoleacetic acid, indole-3-lactic acid, and indole-3-propionic acid) to AhR ligands in the fecal microbiota of patients with T2DM. Enhanced the abundance of *Allobaculum*, *Lactobacillus*, *Akkermansia*, and *Prevotellaceae* in the mice. Reduced intestinal inflammation. Increased concentrations of glycine-conjugated bile acids, DCA, and isoDCA, as well as acetate, propionate, and butyrate in mice.	1. Highlighted roles of bile acids and SCFAs in improving intestinal inflammation and activating AhR ligands. 2. Mechanisms in ENS regulation remain unelucidated.	(Zhou et al., 2024)
a multi-strain probiotic (Lactobacillus sp and Bifidobacterium sp at 30 X 109 CFU) with fructo-oligosaccaride	Patients with Parkinson’s disease and functional constipation (n=55)	For 8 weeks	1. Increased defecation frequency and reduced intestinal transit time. 2. Promoted gastrointestinal peristalsis.	1. A double-blind, randomized, placebo-controlled intervention single center clinical trial. 2. Lacks research on probiotic mechanisms of action.	(Ibrahim et al., 2020)
Synbiotic supplement (combination of Bifidobacterium lactis BB12, Lactobacillus plantarum LP01, and oligofructose-inulin)	Patients aged 18–60 years with functional constipation (n=85)	For 12 weeks	1. Significantly improved stool consistency but failed to effectively increase defecation frequency or improve quality of life.	1. A Randomised, Double-Blind, Placebo-Controlled Trial. 2. Lacked assessment of gut microbiota and effects of different therapeutic dose concentrations.	(Lim et al., 2018)
200 g/day of yogurt with symbiotic added probiotics (Bifidobacterium lactis Bb12, Lactobacillus acidophilus La5, Lactobacillus casei CRL431) and 4g inulin	Healthy males and females (18 to 65 y, BMI 18–35 kg/m2), n=65.	For 15 days	1. Yogurt with or without synbiotics had no effect on gastric emptying time, gastrointestinal transit time, or gastrointestinal symptoms. 2. Synbiotic yogurt resulted in reduced intake of energy, fat, and protein.	1. A double-blind, randomized, crossover study; 2. Study conducted in healthy population; lacks disease specificity.	(Tulk et al., 2013)
Probiotic: *Latilactobacillus sakei Furu* 12019 (L. sakei) Prebiotic: stachyose (ST) Synbiotic: ST + L. sakei	Male ICR mice (6 weeks) with constipation	For 3 weeks	Accelerated GI transit, the synbiotic group was superior to prebiotic or probiotic group. The three groups significantly alter the composition of gut microbiota. Although there are differences in their effects on specific microbial communities, they can all restore the abundance of *Prevotellaceae and Akkermansia*. Increased the expression of GDNF, and decreased NOS, VIP levels in probiotic group. Increased 5-HT levels in synbiotic group. Increased SP, MTL levels in three groups.	1. Although studies showed increased SCFA levels, mechanisms by which SCFAs exert key roles in constipation prevention remain unelucidated. 2. Constipation models exhibit differences in gut microbiota compared with clinical pediatric constipation, notably for Bacteroidota. 3. Demonstrated only the correlation between Prevotella and constipation; failed to investigate causality further.	(Guo et al., 2023b)

Notably, alterations in specific microbiota abundances correlate with ENS restoration and improved GI motility. Studies have shown that increased abundance of *Bifidobacterium, Bacteroides, Prevotella*, and *Akkermansia muciniphila*, or decreased levels of *Dubosiella, Erysipelatoclostridium, Alistipes*, and *Enterococcus faecalis*, may directly contribute to the repairment of the ENS, inhibiting intestinal inflammation and enhancing neurotransmitter synthesis. Kang et al. (2021); Cheng et al., 2023; Lai et al., 2023; Wu et al., 2024; Zhou et al., 2024; Wang et al., 2025) demonstrated that *Lactobacillales* and *Synergistales* exhibit negative and positive correlations with colonic transit time, respectively; however, the regulation of the *Firmicutes/Bacteroidetes* ratio remains highly contentious. Hata et al. (2022) found that an increased *Firmicutes/Bacteroidetes* ratio following *Bifidobacterium* BBG9–1 intervention in patients with diabetic gastrointestinal dysfunction was associated with improved gastrointestinal symptoms, whereas Lee et al. (2024)) reported that reducing this ratio in patients with functional constipation enhanced intestinal peristalsis and shortened colonic transit time. This discrepancy may stem from differences in study populations, and both studies are limited by small sample sizes, making it difficult to exclude confounding effects of baseline microbiota composition, disease duration, and intervention duration. Therefore, larger-scale studies focusing on DGE populations are warranted to further validate the true association between this ratio and GI motility.

Furthermore, current research has significant limitations: mechanistically, although some animal models indicate that microbial therapies can regulate neurotransmitters such as 5-HT and ACh, as well as hormone levels like motilin (MTL), clinical studies show substantial heterogeneity. For example, randomized controlled trials (RCTs) on *Bifidobacterium animalis subsp. lactis* HN019 (referred to as *B. animalis subsp. lactis* HN019) show dose-dependent differences in its effects on colonic transit time: in a triple-blind trial by Waller et al (Waller et al., 2011), high-dose *B. animalis subsp. lactis* HN019 shortened colonic transit time by over 50% in patients with functional constipation (from 49 ± 30 hours to 21 ± 32 hours), while the low-dose group showed only about 30% reduction (from 60 ± 33 hours to 41 ± 39 hours). Similarly, Lai et al. (2023) observed increased defecation frequency and improved straining in chronic constipation patients receiving *B. animalis subsp. lactis* HN019 combined with *Lacticaseibacillus rhamnosus* HN001, though colonic transit time was not assessed. In contrast, a randomized double-blind trial by Ibarra et al. (2018) involving 228 participants found no statistically significant difference in colonic transit time. Both Waller’s and Ibarra’s studies used abdominal X-rays to calculate colonic transit time, ensuring the reliability of their results. In addition, studies in healthy populations (Russo et al., 2011; Tulk et al., 2013) found no evidence that prebiotics or synbiotics promote gastrointestinal peristalsis; some even reported delayed gastric emptying, contradicting findings in models of gastrointestinal dysfunction or diabetes (Lim et al., 2018; Ohtsu et al., 2021). These inconsistencies may arise from strain specificity, dosage variations, treatment duration, and baseline characteristics of participants. Notably, high-quality clinical evidence directly targeting DGE is scarce, with most studies conducted in models of chronic constipation, gastrointestinal motility disorders, or irritable bowel syndrome (IBS). To date, only two double-blind RCTs have demonstrated that *Lactobacillus gasseri* OLL2716 (Ohtsu et al., 2021) and *Bifidobacterium bifidum* G9-1 (Hata et al., 2022) improve delayed gastric emptying and GI symptoms in patients with DGE, but both had small sample sizes, posing significant challenges for clinical translation. Additionally, there is a lack of replicative studies on the same probiotics or prebiotics, as well as limited clinical application. Therefore, future research requires large-scale, multicenter, more replicative RCTs to further validate efficacy, safety, and stability.

At the clinical application level, microbial therapies still face multiple challenges (Suez et al., 2019). Firstly, bioavailability issues: probiotics may experience possible diminution or inactivation as they navigate the hostile milieu in the GI tract, marked by low pH stomach acid and diverse digesting enzymes. Additionally, probiotics exhibit insufficient colonization capacity on gastrointestinal mucosal surfaces, which is further influenced by host microbiota composition and intestinal transit rate. Currently, several innovative strategies are being investigated, including the use of biofilms and nanocoating for “nano armor” (Xu et al., 2022), single-cell technology-based “armored probiotics” (Zhao et al., 2024), targeted delivery systems (Li and Zhang, 2024), and encapsulating probiotics into microcapsules and microspheres using prilling or vibration techniques (D’Amico et al., 2024). These strategies aim to address key issues in the therapeutic use of probiotics by stabilizing probiotic activity, enhancing intestinal colonization, and improving bioavailability. Secondly, safety and long-term effects remain unclear: despite the relative safety of microbial therapies with rare adverse reactions, existing studies suffer from limitations like small sample sizes and short observation periods. Long-term supplementation of single strains may reduce native microbiota diversity and exacerbate intestinal dysbiosis (Elshaghabee et al., 2017). Immunocompromised patients using *Lactobacillus* or *Bacillus* probiotics may develop bacteremia or even septic shock due to sepsis (Corredor-Rengifo et al., 2024). A meta-analysis (Zeng et al., 2024) evaluating 4 RCTs suggested that probiotic supplementation improves glycemic control in T1DM patients without severe adverse events; however, the limited number of RCTs precludes definitive conclusions on the efficacy or safety of microbiome-based therapies, necessitating more RCTs for validation. Furthermore, current interventions predominantly rely on “one-size-fits-all” formulations, neglecting individual variability among patients with DGE. Future efforts should focus on developing personalized regimens integrating host metabolic status and gut microbiota characteristics.

### 5.2 Fecal microbiota transplantation

Fecal microbiota transplantation (FMT), which reestablishes microbial homeostasis by colonizing functional microbiota from healthy donors in the host gut, has demonstrated therapeutic potential in various gastrointestinal diseases (Ooijevaar et al., 2019). In Clostridioides difficile infection (CDI), FMT achieves a clinical symptom resolution rate of 92% (Hvas et al., 2019; Rokkas et al., 2019); in inflammatory bowel disease, systematic reviews report clinical remission rates of 47.5% for Crohn’s disease and 39.6% for ulcerative colitis (Lai et al., 2019); its long-term ameliorative effects on chronic constipation have also been validated, suggesting unique value in repairing intestinal motility disorders (Zhang et al., 2018). More importantly, FMT can modulate the ENS via the “microbiota-neuron” axis: for instance, FMT lowers serum 5-HT and GABA levels and increases dopaminergic neurotransmission to help children with autism who experience digestive issues such as diarrhea, constipation, dyspepsia, and abdominal pain (Li et al., 2021a); in a Parkinson’s mouse model, FMT from healthy donors also improves GI motility by preventing TLR4/TNF-α signaling and neuroinflammation (Sun et al., 2018). These findings support FMT as a potential intervention for neurogenic gastrointestinal motility disorders. In metabolic diseases, FMT regulates 15 key metabolites, including indole-3-propionic acid, reduces leptin levels, and improves insulin sensitivity in patients with T2DM (Zhang et al., 2020; Wu et al., 2022b), indirectly indicating its potential in DGE. However, FMT application in DGE lacks direct clinical evidence, with minimal research addressing core DGE pathologies. The mechanisms by which FMT directly restores the ENS via microbial metabolites also remain unelucidated.

As a special population, diabetic populations face unique risks and challenges in the application of FMT. First, amplified infection risks. Long-term hyperglycemia in diabetes causes immune dysfunction, potentially increasing infection risk with FMT (Campbell et al., 2023). Second, distinct adverse reactions. Beyond common FMT-related symptoms (abdominal pain, diarrhea, nausea) (Aroniadis et al., 2019), patients with diabetes may experience exacerbated intestinal bacterial translocation due to impaired intestinal barrier, triggering systemic inflammation, elevated C-reactive protein, or even bacteremia (Quera et al., 2014). Third, standardization barriers: Unoptimized protocols for stool processing, preservation, and delivery combined with diabetic-specific gut microenvironment alterations compromise donor microbiota engraftment efficiency. Fourth, therapeutic heterogeneity: Zhang et al., 2021a demonstrated that baseline gut microbiota diversity critically determines efficacy, with lower diversity correlating with superior outcomes. Several RCTs have shown that FMT from healthy donors can reverse insulin resistance in diabetes and even preserve islet function in new-onset T1DM (de Groot et al., 2021; Ng et al., 2022; Wu et al., 2022b). However, in mouse models, administration of *Parabacteroides distasonis* accelerated progression of diabetes, primarily due to aberrant immune cross-reactivity and reduced Foxp3^+^ CD4^+^ Treg cells. Thus, given these uncertainties, large-scale multicenter randomized controlled trials are imperative to confirm its long-term efficacy and safety (Zecheng et al., 2023). Addressing these core challenges is essential for translating FMT from theoretical promise to clinically effective therapy for diabetes and DGE.

## 6 Conclusion and perspectives

The dynamic regulatory network formed by gut microbiota and the ENS constitutes a central regulator of intestinal homeostasis, with its dysfunction playing a pivotal role in the pathogenesis and progression of DGE. This review systematically elucidates how the microbiota-ENS axis modulates the function of the ENS through multilevel signaling transduction involving microbial metabolites (SCFAs, BAs), intestinal hormones (GLP-1, CCK, leptin, ghrelin), and neurotransmitters (5-HT, Ach, VIP, NO, GABA), thereby regulating gastrointestinal motility. This mechanism provides a robust theoretical framework for microbiota-targeted therapies in DGE. Current evidence confirms that probiotics, prebiotics, and synbiotics can reshape the structure of gut microbiota and enhance neuroprotection, demonstrating potential to alleviate gastroparesis and improve motility in animal models and limited clinical trials. Although FMT shows advantages in microbiota reconstitution for refractory gastrointestinal diseases, direct evidence for its application in DGE remains insufficient; in particular, the specific association between donor microbiota composition and ENS restoration in patients with DGE requires clarification.

However, clinical translation of microbiome-based therapies still faces multiple challenges. Firstly, causal ambiguity: specific microbiota associated with GI motility in DGE remain unidentified, as most studies are limited to pan-microbial community analyses at the genus or species level. Notably, functional differences between strains of the same species are substantial, and causal validation of the “microbiota-metabolite-nerve” axis is lacking. Secondly, therapeutic heterogeneity: clinical trials exhibit marked heterogeneity, such as the efficacy of probiotics or prebiotics being influenced by the baseline microbiota of host, diabetic duration, and glycemic control, while the scarcity of large-scale, multicenter RCT data hinders the establishment of uniform treatment standards. Thirdly, immune dysfunction may amplify infection risks from probiotics, hyperglycemia-altered microenvironments may reduce the efficiency of microbial colonization, and long-term safety of prolonged interventions lacks longitudinal follow-up data. Fourthly, technical standardization: there is a lack of consensus on viable probiotic delivery, FMT donor screening, and outcome evaluation.

Future research could focus on interdisciplinary breakthroughs (Bober et al., 2018; Wang et al., 2022b): applying spatial transcriptomics and single-cell sequencing to map spatial localization of microbiota-ENS interactions, and decipher interaction sites between specific strains and ENS neuron subsets; engineering probiotics via synthetic biology to achieve precise regulation of neurotransmitters and targeted ENS; establishing a DGE microbiota typing system based on multi-omics integration (genomics, metabolomics, neuroelectrophysiology) to guide personalized interventions; exploring combined strategies of microbial therapies with hypoglycemic agents to balance glycemic control and gastrointestinal motility improvement.

Through refinement of mechanism, personalization of intervention strategies, and scaling of clinical evidence, microbiome research is expected to advance from laboratory to clinical practice, ultimately providing DGE patients with novel therapeutic regimens that integrate neural repair, metabolic regulation, safety, and tolerability, thereby tangibly improving their gastrointestinal function and quality of life.

## Glossary

DGE: Diabetic gastroenteropathy
GI: gastrointestinal
T2DM: type 2 diabetes mellitus
GSRS: Gastrointestinal Symptom Rating Scale
GCSI: Gastroparesis Cardinal Symptom Index
FDA: Food and Drug Administration
ENS: enteric nervous system
CNS: central nervous system
ICCs: interstitial cells of Cajal
ECCs: enterochromaffin cells
EGCs: enteric glial cells
EECs: enteroendocrine cells
IBS: irritable bowel syndrome
IBS-D: diarrhea-predominant
IBS-C: constipation-predominant
IBD: inflammatory bowel disease
SCFAs: short-chain fatty acids
FMT: fecal microbiota transplantation
AHR: aryl hydrocarbon receptors
TLRs: Toll-like receptors
GDNF: glial cell line-derived neurotrophic factor
LPS: lipopolysaccharides
NF-κB: nuclear factor kappa-B
HDAC: inhibiting histone deacetylase
TMA: trimethylamine
SERT: serotonin transporter
PYY: peptide YY
Kyn: kynurenine
DCA: deoxycholic acid
LCA: lithocholic acid
NO: nitric oxide
CGRP: calcitonin gene-related peptide
IBAT: ileal bile acid transporter
CCK: cholecystokinin
GIP: glucose-dependent insulinotropic polypeptide
TNF-α: tumor necrosis factor-α
GHSR1a: growth hormone secretagogue receptor 1a;Ach:acetylcholine
GABA: γ-aminobutyric acid
VIP: Vasoactive Intestinal Peptide
ChAT: choline acetyltransferase
NOS: nitric oxide synthase
nNOS: neuronal NOS
GAD: glutamate decarboxylase
SS: somatostatin
MTL: motilin
SP: substance P
FOS: fructooligosaccharides
GOS: galactooligosaccharides
WGTT: whole-gut transit time.
